# *In silico* identification of phytocompounds derived from *Glycyrrhiza glabra* as potential inhibitors of actin assembly-inducing protein in *Listeria monocytogenes*: a virtual screening and molecular dynamics study

**DOI:** 10.3389/fbinf.2026.1822250

**Published:** 2026-04-28

**Authors:** Deepasree K., Sudha Ramaiah

**Affiliations:** 1 Medical and Biological Computing Laboratory, School of Bio Sciences and Technology (SBST), Vellore Institute of Technology (VIT), Vellore, Tamil Nadu, India; 2 Department of Bio-Sciences, School of Bio Sciences and Technology (SBST), Vellore Institute of Technology (VIT), Vellore, Tamil Nadu, India

**Keywords:** *ab initio* modelling, Lipinski’s rule, liquorice, phytocompounds, virtual screening, virulence protein

## Abstract

**Introduction:**

Natural compounds present in medicinal plants have made significant contributions to the field of drug development due to their diverse therapeutic properties. One such crucial application of the phytocompounds is to suppress the survival of pathogenic microbes that withstand the current treatment regimens.

**Methods:**

Following an *in silico* methodology, the primary goal of this study was to understand the potential inhibitory action of 106 phytocompounds of Liquorice (*Glycyrrhiza glabra*) on the virulence protein ActA present in *Listeria monocytogenes*. The ActA protein was modelled initially and virtual screening was further performed to confirm the potential candidates.

**Results:**

Xambioona and Licoisoflavone B exhibited good binding affinity values of −10.4 kcal/mol and −8.7 kcal/mol with the AlphaFold model of ActA protein, respectively.

**Discussion:**

Molecular dynamics (MD) simulation for a timescale of 100 ns and binding free analysis revealed Licoisoflavone B to be a promising phytocompound due to its overall conformational stability.

## Graphical Abstract

**Figure:**
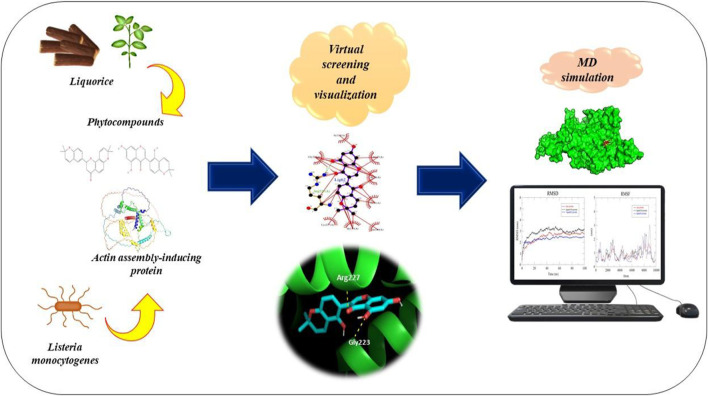

## 1 Introduction

Plants have always played a major role in medicine to cure and treat various diseases since ancient times. Phytocompounds or other plant-derived metabolites act as the prime source of natural molecules that could facilitate the development of novel drugs due to their unique chemical structures and excellent biological activity (Tu et al., 2021; Lordani et al., 2018). Some of the important plant phytochemicals or bioactive compounds that are widely used in modern medicine include terpenoids, flavonoids, tannins, alkaloids, phenolic compounds, saponins and others whose utilization has led to improved treatment and reduced side effects (Sharma et al., 2018). *Glycyrrhiza glabra* commonly known as liquorice or sweet wood in English is one of the ancestral herbs belonging to the family *Fabaceae* which is used both as a flavouring agent and an antidote from the birth of Ayurveda. This plant is named differently around numerous parts of the world like Helichrysum petiolare (in South Africa), Gancao (in China) and as Mulaithi (North India), iratimadhuram (Malayalam), atimaduram (Tamil), atimaddhura (Kannada) in India (Kaur et al., 2013). It is an “essential herbal medicine” that is mostly found in the Mediterranean and specific regions of Asia. Nowadays, it is also commonly grown in Russia, Europe and China. The *Glycyrrhiza* genus is derived from the Greek words *‘glykos’* (sweet) and ‘*rhiza’* (root) and constitutes over 30 species that are distributed globally (Jiang et al., 2020; Batiha et al., 2020; Pastorino et al., 2018; Murray, 2020). For the past few years, researchers from across the world have been actively working on determining the pharmacological activities of liquorice that makes it exceptional from other medicinal plants. As a result, scientists have discovered a wide range of pharmacological properties displayed by this perennial herb some of which include antiulcer, antitumor, antiallergic, anti-inflammatory, antimicrobial activities, memory-enhancing effects and protection of skin. The organ systems with potential benefits include the nervous, cardiovascular, alimentary, respiratory and endocrine systems (Pandey, 2017). The active constituents of liquorice are the major contributors to these therapeutic effects (Zadeh et al., 2013; Thakur and Raj, 2017; Kumar and Dora, 2017).

Resistance of pathogens to antibiotics is a serious concern that has drastically affected both public health and economic sectors. *Listeria monocytogenes,* a gram-positive food-borne pathogen is an etiological agent that causes listeriosis, a severe invasive disease that could be life-threatening to vulnerable individuals including pregnant women, newborns, older adults and those with compromised immune systems (Coelho et al., 2019; Matereke and Okoh, 2020; Lopes-Luz et al., 2021; Disson et al., 2021). The ability of this bacteria to adapt and replicate in extreme environmental stress conditions has ultimately affected food processing industries since the pathogen has become a common food contaminant. Among the several virulence factors possessed by *Listeria*, actin assembly-inducing protein (ActA) is the main focus of this study as it is also one of the crucial virulent proteins that aid the pathogen to withstand stress conditions (Bucur et al., 2018; Swaminathan and Gerner-Smidt, 2007). This protein has a signal sequence, a central proline-rich repeat domain and a C-terminal hydrophobic end that is followed by positively charged residues to maintain the protein within the bacterial membrane (Microbiology et al., 1993). This protein is required for the intracellular motility, cell-to-cell spread and dissemination of the pathogen in the infected host. These activities are facilitated through actin recruitment and polymerisation (Welch, 1998; Pistor et al., 1994; Brundage et al., 1993; Pistor et al., 1995).

Since the protein lacks a unique structure, the modelling of ActA was performed. The antimicrobial effects of some of the major phytocompounds of liquorice have previously been studied on several pathogens. However, their impact on this virulence factor of *Listeria* has never been explored to date. Thus, the main objective of this work is to utilize *in silico* techniques like virtual screening and simulation to explore the therapeutic potential of different phytocompounds of liquorice in suppressing the activity of modelled protein ActA of *Listeria monocytogenes*.

## 2 Materials and methods

### 2.1 Protein modelling and validation

Initially, the protein sequence of ActA (Accession No. P33379) was retrieved from the UniProt database (https://www.uniprot.org/) (UniProt Consortium, 2019). The physicochemical characteristics of ActA protein such as molecular weight, estimated half-life, atomic composition, theoretical pI, amino acid composition, aliphatic index, instability index and grand average of hydropathicity (GRAVY) were estimated from its UniProt query sequence with the help of ProtParam tool (https://web.expasy.org/protparam/) (Gasteiger et al., 2025). After performing a BLASTp analysis, it was found that the query sequence did not display any similar templates. Due to this unavailability of a template protein, the query sequence was submitted to AlphaFold Colab (a Google DeepMind software) and RoseTTAFold (an *ab initio* modelling tool) to predict the three-dimensional structure (Jumper et al., 2021; Mirdita et al., 2022; Baek et al., 2021). The topmost models generated from these servers were further subjected to energy minimisation and refinement using Swiss PDB Viewer and Galaxy Refine Server (Guex and Peitsch, 1997; Heo et al., 2013).

**FIGURE 1:**
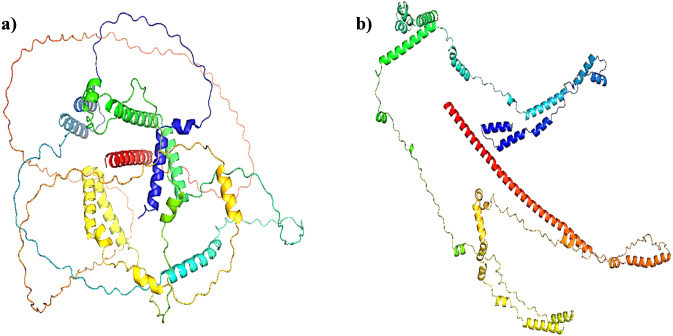
ActA models generated from: **(a)** AlphaFold Colab and **(b)** RoseTTAFold.

The quality of the models was finally validated utilizing various servers. Ramachandran Plot (http://www.ebi.ac.uk/thornton-srv/databases/pdbsum/) helps to understand the stereochemical quality of the 3D structures (Laskowski et al., 2018). ERRAT (https://saves.mbi.ucla.edu/) was used to analyse the overall quality of the two models produced from AlphaFold and RoseTTAFold (Colovos and Yeates, 1993) while ProSA-web was utilized to determine the potential errors in the 3D models (Wiederstein and Sippl, 2007). Figure 1 represents the models obtained from the respective modelling software. Both models were then confirmed and used for further study.

### 2.2 Preparation of protein

Before virtual screening, the modelled proteins were prepared in AutoDock Tools (ADT) 1.5.7 software (Morris et al., 2009). All the water molecules were removed at the beginning and this was followed by the addition of polar hydrogens and Kollman charges to the modelled targets. The prepared proteins were finally saved in pdbqt format.

### 2.3 Collection and preparation of phytocompounds of liquorice

A total of 106 phytocompounds from various parts of *Glycyrrhiza glabra* which included the leaf, stem, root, and other regions were gathered from the IMPPAT (Indian Medicinal Plants, Phytochemistry And Therapeutics) database (Mohanraj et al., 2018). This curated database provides information on the diverse phytochemicals of various Indian medicinal plants along with their therapeutic properties. The 3D conformers of the selected phytocompounds (ligands) were then downloaded from the PubChem database (https://pubchem.ncbi.nlm.nih.gov/) in SDF format (.sdf) (Kim et al., 2023). The ligands were then energy minimized with the help of Avogadro software that utilized the steepest descent algorithm at a maximum of 500 steps and convergence of 1x 10^−7^ (Hanwell et al., 2012). Using the Open Babel GUI programme (version 2.3.1), the selected ligands were eventually converted to pdbqt formats (O’Boyle et al., 2011).

### 2.4 Virtual screening of phytocompounds against ActA models and visualization

Blind docking was preferred for screening all the 106 ligands against the two ActA protein models and the AutoDock Vina program was employed for the same. Virtual screening plays a crucial role in the drug discovery process by identifying potential drug-like compounds (also called as hits) that have a greater affinity to bind with particular biological targets (protein receptors or enzymes). Thus, this computational technique filters out all other compounds with low binding affinities to the target, making it significantly more effective than the experimental approach (Walters et al., 1998; Rehman et al., 2019). AutoDock Vina uses an effective optimization algorithm which is based on a unique scoring function for calculating the protein-ligand affinities and a new search algorithm for predicting the possible binding modes (Jaghoori et al., 2016). In this study, the X, Y and Z dimensions were set to 126 points with a spacing of 1 Å such that the grid box covered the entire protein models while the number of modes were set to 10. The grid boxes of the predicted models were centered at x = 7.104, y = −0.677, z = 4.209 (for AlphaFold model) and x = 1.707, y = 37.121 and z = 1.396 (for RoseTTAFold model).

**FIGURE 2:**
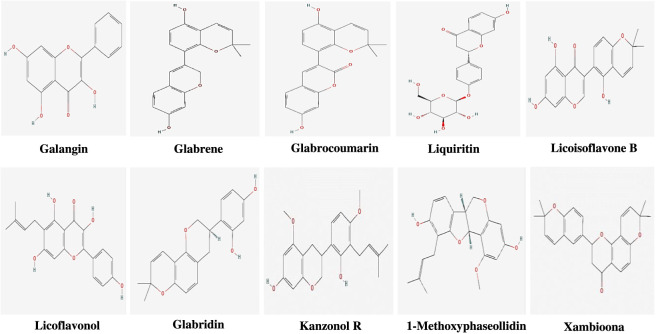
Structures of top 10 phytocompounds of Liquorice.

Following virtual screening, the drug-likeliness of all 106 compounds was evaluated based on Lipinski’s rule of five to assess their oral bioavailability and the PubChem database was used for the same. This rule states that an orally active drug-like compound should have a molecular weight of less than 500 g/mol, no more than 5 hydrogen bond donors, no more than 10 hydrogen bond acceptors and an octanol-water partition coefficient (log P) not greater than 5 (Benet et al., 2016). Thus, the best 10 compounds that were Lipinski compliant and also displayed negative binding affinity values greater than −5.5 kcal/mol were taken into account. Moreover, the binding site pocket geometry of the protein models were determined using CASTpFold server (Ye et al., 2024). Table 1 and Figure 2 list the top 10 phytocompounds of liquorice that satisfied Lipinski’s criteria without any violations and also exhibited good binding affinity values with both protein models. To better understand the two-dimensional and three-dimensional interactions involved in the binding process, all 10 protein-ligand complexes were further visualized in Ligplot+ and PyMOL after virtual screening (Laskowski and Swindells, 2011; Yuan et al., 2017). Furthermore, the key binding site residues were mapped on the best protein model of ActA using UCSF ChimeraX (Meng et al., 2023).

**TABLE 1:** Top 10 phytochemicals of Liquorice that obey Lipinski’s rule.

Ligand No.	Phytocompounds	Molecular weight (g/mol)	Hydrogen bond donors (HBD)	Hydrogen bond acceptors (HBA)	Log P
2	Galangin	270.24	3	5	2.3
27	Glabrene	322.4	2	4	3.6
34	Glabrocoumarin	336.3	2	5	3.8
54	Liquiritin	418.4	5	9	0.4
62	Licoisoflavone B	352.3	3	6	3.6
63	Licoflavonol	354.4	4	6	3.8
71	Glabridin	324.4	2	4	3.9
78	Kanzonol R	370.4	2	5	4.8
82	1- Methoxyphaseollidin	354.4	2	5	4.2
106	Xambioona	388.5	0	4	4.8

### 2.5 Molecular dynamics (MD) simulation and binding free energy estimation

Based on the virtual screening results, MD simulations were carried out for two protein-ligand complexes and the apo form of ActA protein for 100 ns. GROMACS (2022.3 version) package with Ubuntu 20.04.5 LTS OS was used for the same (Bauer et al., 2022). The complexes of Xambioona and Licoisoflavone B with ActA (AlphaFold model) were subjected to simulation in order to interpret the stability of the docked protein-ligand complexes over the course of 100 ns time scale. Initially, the protein and ligand topologies were generated using the CHARMM36 force field of GROMACS and CGenFF (version 4.6) server (Huang et al., 2017; Vanommeslaeghe et al., 2010). This was followed by the solvation of protein-ligand complexes and apo form in a cubic box to mimic the biological environment. Na^+^/Cl^−^ ions were then added to neutralize the system. Using the steepest descent algorithm and verlet cut-off scheme, energy minimization of the system was performed at 10 kJ/mol to relax possible steric clashes developed during topology file creation. Post energy minimization, the systems underwent NVT and NPT equilibration for 100 ps using the Berendsen thermostat method at 300K and 1 bar pressure maintained by the Parrinello-Rahman method (Andersen, 1980; Berendsen et al., 1984). Moreover, LINCS (Linear constraint solver for molecular simulations) algorithm and the Particle-Mesh Ewald method were used to evaluate the constraints imposed by hydrogen atoms and also to determine the electrostatic interactions involved in the system (Petersen, 1995). The coordinates were stored for every 2fs and 100 ns MD run was finally initiated after equilibration. Furthermore, the root mean square deviation (RMSD), root mean square fluctuation (RMSF), radius of gyration (Rg) and hydrogen bond formation in the systems were evaluated using analytical tools like gmx rmsd, gmx rmsf, gmx gyrate and gmx hbond. Plots for all of these parameters were generated using the Xmgrace software (Turner, 2005).

The overall binding free energies of the best protein-ligand complexes were determined through MM/PBSA (Molecular Mechanics/Poisson-Boltzmann Surface Area) analysis utilizing the *gmx_MMPBSA* program in the GROMACS suite (Valdés-Tresanco et al., 2021). The total binding free energy (ΔG_bind_) along the 100 ns MD trajectory was determined based on the equation:

$$\Delta G_{\text{bind}}=G_{\text{complex}}-G_{\text{protein}}-G_{\text{ligand}}$$

where G_protein_ and G_ligand_ are free energies of the target protein and ligands while G_complex_ represents the free energy of the protein-ligand complex.

## 3 Results

### 3.1 Primary sequence analysis

The physicochemical properties of the ActA protein sequence (obtained from the UniProt database) were computed using the Protparam server (Table 2). With a length of 639 amino acids, the protein displayed a molecular weight of 70.34 kDa and a theoretical isoelectric point (pI) of 5.01. This protein comprised of 3062 carbon atoms, 4930 hydrogen atoms, 846 nitrogen atoms, 1021 oxygen atoms and 13 sulfur atoms contributing to a total number of 9872 atoms. Glutamate (E) made up a significant portion of the protein with a maximum composition of 11.4% followed by 9.5% of serine (S). 9.1% and 8.9% of Lysine (K) and Proline (P) were also present in the protein with the least contribution of 0.2% by cysteine (C). Moreover, a total of 108 negatively charged and 85 positively charged amino acid residues were also observed. The instability index, grand average of hydropathicity (GRAVY) and aliphatic index were found to be 58.46, −0.797 and 67.72. Generally, a predicted instability index value ≤40 indicates that a protein is stable in a test tube at normal temperature. Here, ActA protein exhibited a value greater than 40 indicating that the protein may be unstable at this temperature. The aliphatic index value, which measures the relative volume occupied by aliphatic side chains in a protein, further suggests the thermal stability of the ActA protein. Based on the displayed negative GRAVY value, the protein was inferred to be hydrophilic in nature. The estimated half-life was found to be 30 h (mammalian reticulocytes, *in vitro*), >20 h (yeast, *in vivo*) and >10 h (*E. coli*, *in vivo*) suggesting that ActA protein disappears faster post its synthesis in the cell.

**TABLE 2:** Physico-chemical parameters of ActA protein sequence.

Accession No.	No. of amino acids	Molecular weight (kDa)	Theoretical pI	Total no. of atoms	No. of negatively charged amino acids	No. of positively charged amino acids	Instability index	Aliphatic index	GRAVY
P33379	639	70.34	5.01	9872	108	85	58.46	67.72	−0.797

### 3.2 Validation of AlphaFold colab and RoseTTAFold models

The finalisation of reliable protein models depends on protein validation, which is required to assess the quality of generated models.

#### 3.2.1 Ramachandran Plot

**FIGURE 3:**
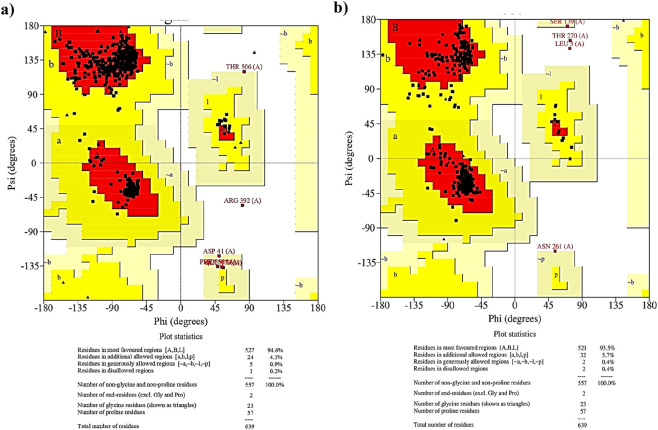
Ramachandran plots obtained for **(a)** AlphaFold Colab model and **(b)** RoseTTAFold model. Red, yellow, pale yellow and white colour regions indicate the most favoured, allowed, generously allowed and disallowed regions in the plot.

Both the models of ActA were initially validated using the generated Ramachandran plots. This plot shows the distribution of torsional angles (ф and ѱ) of amino acid residues present in a peptide thereby providing an insight into the number of residues contained in the ‘allowed’ and ‘disallowed’ regions of the plot (Laskowski et al., 2013). Figure 3 represents the Ramachandran plots of the AlphaFold Colab and RoseTTAFold models. The percentage of residues in the most favoured regions, additionally allowed regions and generously allowed regions were estimated to be 94.6%, 4.3% and 0.9% for the AlphaFold model while only 0.2% of residues were present in the disallowed regions. Likewise, 0.4% of residues were in the disallowed and generously allowed regions for the RoseTTAFold model along with 93.5% and 5.7% amino acid residues in its most favoured and additional allowed regions. A model is deemed to be of good quality if it has greater than 90% residues in the most favoured regions. In this case, AlphaFold model displayed greater percentage of residues in the most favoured regions suggesting the model to be more reliable than RoseTTAFold model.

#### 3.2.2 ERRAT

**FIGURE 4:**
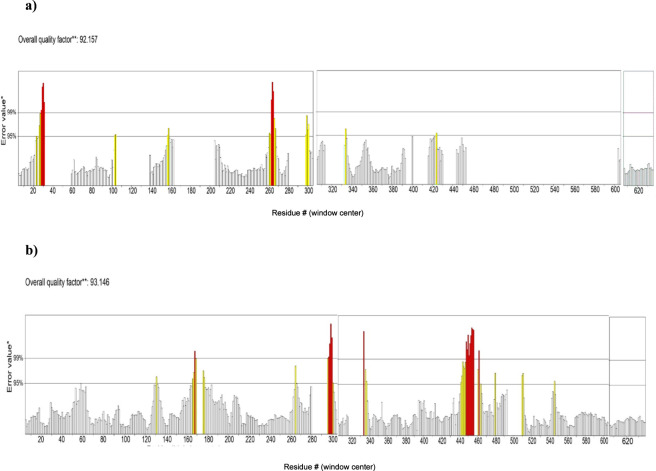
ERRAT plots of **(a)** AlphaFold Colab model and **(b)** RoseTTAFold model. Bars indicate the non-bonded interactions of the residues where red and yellow bars represent the error values of residues greater than 99% and 95% of the confidence level.

The SAVESv6.0 server was used to produce ERRAT scores for further validation of protein models. ERRAT predicts the overall quality of a protein model based on a value referred to be the “overall quality factor” (Patel et al., 2020). Typically, the statistics of non-bonded interactions between residues in the protein models are analyzed using the ERRAT plot. Good quality structures are indicated by higher ERRAT scores, which are often above 50 (Omar et al., 2018). Figure 4 displays the ERRAT plots of the AlphaFold colab and RoseTTAFold models, respectively. The values for the overall quality factor of Alphafold colab and RoseTTAFold models were 92.157 and 93.146 revealing that the modelled proteins are of good quality.

#### 3.2.3 Z-score and energy profile analysis

**FIGURE 5:**
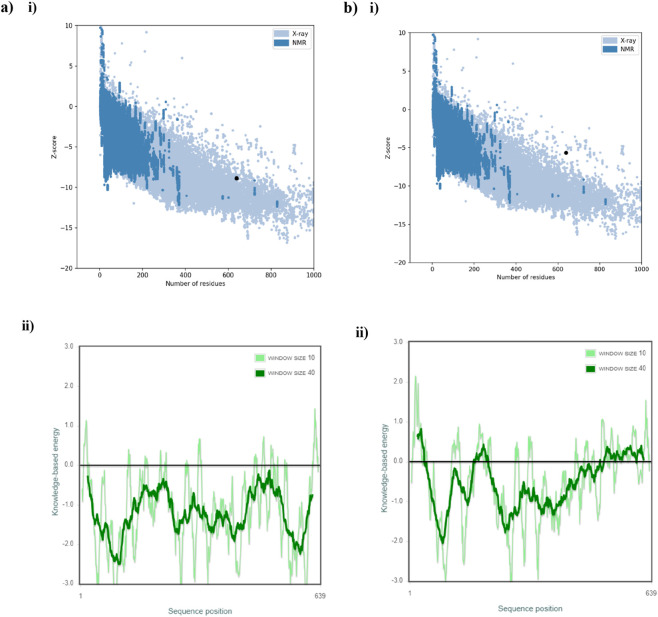
Z-score plot and energy profiles of **(a)** (i, ii) AlphaFold Colab model and **(b)** (i, ii) RoseTTAFold model. The black dots represent the Z-scores of the models. In the energy profiles, the negative values indicate the best region of the predicted models.

ProSA-web server was the next tool employed for protein validation. Z-score is an indication of the overall model quality and also determines if the model falls within the range of values often observed in the experimentally determined native proteins of similar size (Dhanavade et al., 2013). It also determines how far the total energy of the model deviates from an energy distribution based on random conformations. Here, the Z-score values of both AlphaFold Colab and RoseTTAFold models were found to be −8.89 and −5.66 which lies within the acceptable range of −10 to 10. Research has also shown that a more reliable model is indicated by a greater negative Z-score (Gupta et al., 2013). Hence, this analysis suggests that AlphaFold model is a more reliable one than the RoseTTAFold model. The Z-score and energy plots of both models are shown in Figure 5.

### 3.3 Virtual screening analysis

All the 106 phytocompounds of Liquorice were screened against the 2 predicted models and the binding affinities of the compounds to each of the modelled proteins were noted (Supplementary Files S1, S2). The top 10 ligands that displayed high binding affinity values with any one of the modelled proteins and also obeyed Lipinski’s rule were chosen for further analysis. According to the virtual screening results, it was found that the compounds showed higher binding affinity values with the AlphaFold model than with the RoseTTAFold model.

Moreover, AlphaFold model displayed a pocket volume of 211446.13 Å^3^ while RoseTTAFold model had an enlarged pocket volume of 286691 Å^3^. These values could suggest that the less enlarged pockets in AlphaFold model contributed to its more compact binding with the ligands. Therefore, the active site residues and the interactions involved between each of the 10 ligands and the AlphaFold model were examined considering the better stereochemical quality, pocket geometry of the AlphaFold model and better binding affinity of the ligands to the predicted AlphaFold model. Table 3 provides detailed information about the relative binding affinities of the top 10 phytocompounds with both the models along with the amino acid residues involved in the binding of each of the 10 ligands with the AlphaFold model.

**TABLE 3:** Virtual screening results of the top 10 phytocompounds that obeyed Lipinski’s rule and also displayed good docking scores with modelled proteins.

Ligand No.	Ligand name	Binding affinity (kcal/mol)		Interacting amino acid residues (Ligand-AlphaFold model)	
		AlphaFold colab	RoseTTAFold	H-bonds	Hydrophobic interactions
2	Galangin	−7.8	−6.4	Arg227	Phe216, Lys220, Ile20, Gly223, Ala17, Thr16, Val13, Ile219
27	Glabrene	−7.9	−6.6	Gln208	Ile219, Phe216, Val215, Phe212, Phe211, Arg8, Val12, Arg5, Ala9, Pro210
34	Glabrocoumarin	−8.1	−6.5	Gln208	Ile219, Val215, Pro210, Arg8, Arg5, Phe211, Val12, Phe216, Phe212
54	Liquiritin	−8.5	−6.2	Val13, Lys220, Asp231	Ile219, Thr16, Lys224, Gly223, Ile20, Lys237, Ile230, Thr21, Ile240, Ala17
**62**	**Licoisoflavone B**	**−8.7**	−6.7	Arg227	Ile219, Ala17, Ile20, Thr21, Ile240, Ile230, Lys237, Gly223
63	Licoflavonol	−7.8	−6.4	Arg227	Thr21, Ile20, Gly223, Lys220, Phe216, Val13, Ile219, Thr16, Val226
71	Glabridin	−8.3	−7.0	Asn18	Gly622, Phe14, Met618, Thr21, Arg227, Ile230, Asp231, Leu619, Ala17
78	Kanzonol R	−7.8	−6.4	-	Pro210, Phe216, Val215, Phe6, Phe212, Ala9, Phe211
82	1- Methoxyphaseollidin	−8.2	−5.8	-	Phe212, Phe6, Arg5, Phe211, Ala9, Pro210, Val12, Phe216, Val215
**106**	**Xambioona**	**−10.4**	−8.0	-	Phe212, Phe6, Ala9, Phe211, Val12, Pro210, Val215, Phe216, Ile219

The bold compounds are the ones that displayed the best binding affinities.

The aforementioned binding affinity values reveal how efficiently and competitively a ligand interacts with the target protein. A high negative binding affinity value may also indicate the ability of the compound to inhibit the virulence protein (Majeed et al., 2021; Ghosh et al., 2025). The top 10 phytocompounds of liquorice that displayed good scores were noted to be Galangin, Glabrene, Glabrocoumarin, Liquiritin, Licoisoflavone B, Licoflavonol, Glabridin, Kanzonol R, 1- Methoxyphaseollidin and Xambioona, respectively. All these compounds showed negative binding affinity values greater than the threshold value of −5.5 kcal/mol. The scores obtained for ligand-RoseTTAFold model complexes ranged from −5.8 to −8.0 kcal/mol. Similarly, the scores ranged from −7.8 to −10.4 kcal/mol for ligand-AlphaFold model complexes. Thus, the AlphaFold model showed promising virtual screening results with the phytocompounds compared to the RoseTTAFold model. Subsequently, as the AlphaFold model showed good affinity values, the key interactions and amino acid residues involved in the ligand binding process were analyzed.

**FIGURE 6:**
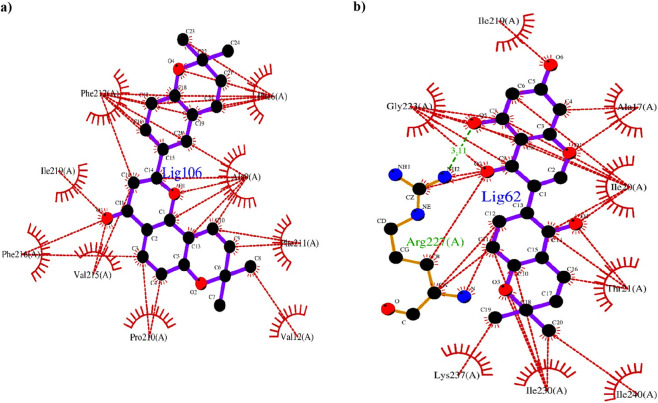
Two-dimensional view of binding interactions between ligands and amino acid residues of predicted ActA model. **(a)** Xambioona (ligand 106) interacting with the modelled protein. **(b)** Interaction of Licoisoflavone B (ligand 62) with the protein. Red dotted lines represent the hydrophobic interactions and the green dotted lines indicate the hydrogen bonds.

**FIGURE 7:**
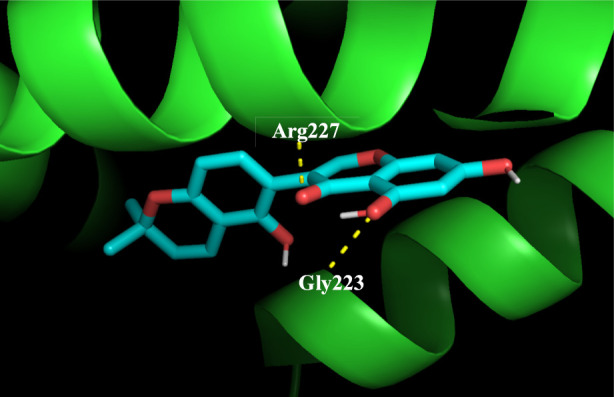
Three-dimensional view of Licoisoflavone B interacting with ActA protein. Yellow dotted lines represent the hydrogen bonds formed between the complex. The 3D visualization revealed 2 hydrogen bonds (between ligand-Arg227 and ligand-Gly223) in this case.

Among the top 10 phytocompounds, Xambioona (ligand 106) exhibited an exceptionally high docking score of −10.4 kcal/mol. This compound did not form hydrogen bonds with any of the amino acid residues in the protein model but had 29 hydrophobic interactions with amino acid residues which included Phe212, Phe6, Ala9, Phe211, Val12, Pro210, Val215, Phe216 and Ile219 (Figure 6a). Licoisoflavone B (ligand 62) was identified to be the next best compound to have displayed a binding affinity value of −8.7 kcal/mol. This ligand formed 1 hydrogen bond and 6 hydrophobic interactions with Arg227. The remaining 22 hydrophobic interactions were to amino acid residues Ile219, Ala17, Ile20, Thr21, Ile240, Ile230, Ly237 and Gly223 (Figure 6b). The hydrogen bond was created at a distance of 3.11 Å between NH-2 of Arg227 and functional group O5 of ligand 62. The 3D view of hydrogen bonding between ligand 62 and ActA model is depicted in Figure 7. This variation in hydrogen bond numbers can be related to the discrepancies in the angular and distance cutoffs that are employed to detect the hydrogen bonds. In 2D depiction, hydrogen bond interactions in close proximity to threshold values may not necessarily be included due to the rigid geometric constraints. However, in 3D visualization these interactions may be captured. So, Gly223 upon 3D visualization could possibly indicate a weak interaction. Following Licoisoflavone B, Liquiritin (ligand 54) possessed a binding affinity value of −8.5 kcal/mol wherein it formed 3 hydrogen bonds with Val13, Lys220 and Asp231. The hydrogen bonds were formed at distances 3.21 Å (between O of Val13 and O8 of ligand 54), 2.88 Å (between O of Lys220 and O9 of ligand 54) and 2.97 Å (between OD1 of Asp231 and O3 of ligand 54) respectively. Furthermore, this ligand showed 9 hydrophobic interactions with Ile230, Thr21, Ile240, Ala17, Ile219, Thr16, Lys224, Gly223, Ile20, and Lys237. Phytocompounds Glabridin (ligand 71), 1-Methoxyphaseollidin (ligand 82), Glabrocoumarin (ligand 34) and Glabrene (ligand 27) exhibited binding affinity values of −8.3, −8.2, −8.1 and −7.9kcal/mol. Among these four compounds, both Ligand 27 and ligand 34 formed 1 hydrogen bond with Gln208 while ligand 71 displayed 1 hydrogen bond with Asn18. Ligand 82 only had hydrophobic interactions. The hydrogen bond distances were particularly 3.23 Å (between OE1 of Gln208 and O4 of ligand 27), 3.27 Å (between OE1 of Gln208 and O5 of ligand 34) and 2.88 Å (between OD1 of Asn18 and O4 of ligand 71). The least affinity value of −7.8 kcal/mol was shown for Galangin (ligand 2), Licoflavonol (ligand 63) and Kanzonol R (ligand 78). Thus, Xambioona and Licoisoflavone B were chosen for further studies because of their best affinity values. Additionally, strong binding of ligand-protein complexes were observed even in the absence of hydrogen bonds suggesting that hydrophobic interactions equally play an inevitable role in the binding process.

Some of the amino acid residues that frequently occurred during the interaction with the 10 phytocompounds were notably Phe216, Ile219, Phe211 and Pro210. Thus, these residues present in the ActA predicted model could possibly play a vital role in making the virulence protein inactive on its binding with the potential compounds mentioned earlier.

### 3.4 Structural mapping of key binding site residues in ActA

**FIGURE 8:**
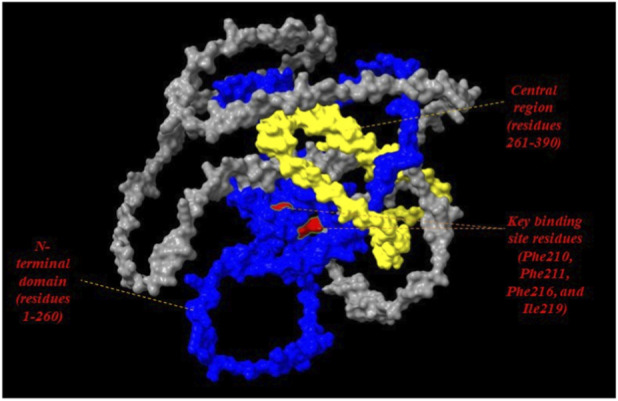
Structural mapping of crucial binding site residues in the AlphaFold model of ActA. The virulence protein is represented as a cartoon where the functional regions are depicted in blue (N-terminal domain), yellow (central region) and red (key residues: Phe210, Phe211, Phe216, and Ile219).

The key interacting residues Phe210, Phe211, Phe216, and Ile219 were identified to form a spatially localized cluster in the 210–220 region (Figure 8). These residues are mostly surface exposed and positioned adjacent to the N-terminal domain, which is considered functionally involved in actin nucleation. Their localization implies closeness to areas that may be interacting with host proteins such as the Arp2/3 complex and VASP. This geometrical layout of the predicted binding site lends credence to the functional significance of the site and its possible importance in protein-protein interactions.

### 3.5 Molecular dynamics simulation analysis

Two independent MD simulations were performed for the apo form of modelled ActA protein (AlphaFold colab model), ActA – Xambioona (lig.106) complex and ActA – Licoisoflavone B (lig.62) complex for a time scale of 100 ns. Xambioona and Licoisoflavone B complexes with ActA protein were chosen since both the compounds exhibited good binding affinity values. The 100 ns simulations were carried out to check if the complexes changed in any way over the specific time period with regard to their stability, conformation and interactions with amino acid residues (Joshi et al., 2021; Surti et al., 2020). The RMSD, RMSF, Rg and hydrogen bonding between the complexes were then determined as a function of time using the generated MD trajectories.

#### 3.5.1 Root mean square deviation (RMSD)

**FIGURE 9:**
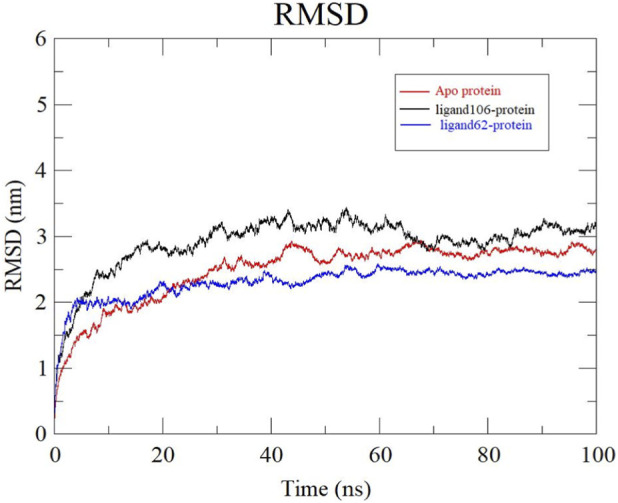
RMSD plots of Apo protein (red), ActA-Xambioona complex (black) and ActA-Licoisoflavone B complex (blue). Xambioona is represented as Ligand 106 and Licoisoflavone B is represented as ligand 62.

The stability of the apo protein as well as the protein-ligand complexes were computed based on the RMSD values. RMSD helps to understand how much the protein has deviated from its initial structural conformation upon ligand binding (Mani et al., 2023; Rampogu et al., 2022). The backbone RMSD plots of apo protein, ActA-ligand106 and ActA-ligand62 complexes were examined and compared. Apo protein moderately fluctuated and stabilized around 50 ns at an RMSD of 2.66 ± 0.15 nm. Conversely, the ligand106-protein complex had greater deviations and displayed an RMSD of 2.85 ± 0.21 nm implying relatively less stability. However, the ActA-ligand62 complex was less deviating and gained stability after 20 ns at an RMSD of 2.41 ± 0.10 nm. Thus, according to these results, the ActA-Licoisoflavone B complex was found to be more stable compared to the apo protein and ActA-Xambioona complex (Figure 9). This indicates that the ActA protein attained more stability upon the binding of Licoisoflavone B.

#### 3.5.2 Root mean square fluctuation (RMSF)

**FIGURE 10:**
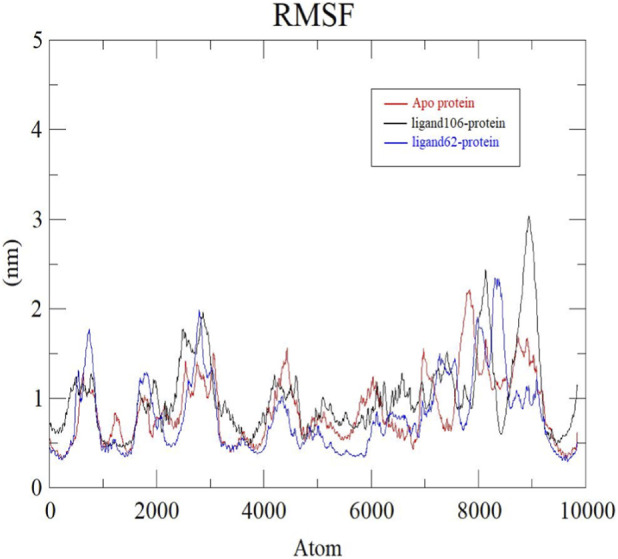
RMSF plots of Apo protein (red), ActA-Xambioona complex (black) and ActA-Licoisoflavone B (blue).

RMSF analysis was done to have a better understanding of the atom fluctuations that occur in the protein during ligand binding. Hence, more flexible regions (loops, turns) of the structure are indicated by high RMSF values whereas the presence of secondary structures (helices, sheets) is confirmed by lower RMSF values (Bhatt et al., 2021; Shahlaei et al., 2011; Krushna et al., 2017). Figure 10 depicts the backbone RMSF plots of apo protein, ActA-ligand106 and ActA-ligand62 complexes. The flexibility of the apo protein was moderate with an average RMSF of 0.96 ± 0.20 nm, and higher fluctuations were observed in loop regions especially around atoms 2000–3000 and 7500–8200. On the other hand, the ligand106-protein complex had much greater fluctuations, particularly at the area covering atoms 8500–9000, contributing to an average RMSF value of 2.89 ± 0.35 nm. The ligand62-protein complex displayed relatively reduced fluctuations and exhibited an average RMSF of 0.75 ± 0.16 nm, suggesting that there was increased structural rigidity on ligand binding.

**FIGURE 11:**
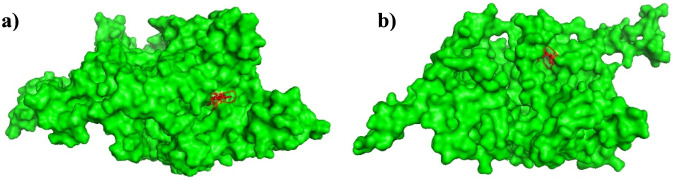
100 ns trajectory analysis of **(a)** Xambioona-ActA and **(b)** Licoisoflavone B-ActA complexes. The ligands (red region) stayed bound to the virulence protein (green region) by the end of 100 ns simulation.

There occurred no significant dissociation events and the ligands remained bound to the ActA protein throughout the 100 ns MD simulation. Trajectory analysis of both the complexes confirmed that the ligands Xambioona and Licoisoflavone B retained their overall binding and significant contacts over time, indicating the dynamic stability of the complexes (Figures 11a,b).

#### 3.5.3 Radius of gyration (Rg)

**FIGURE 12:**
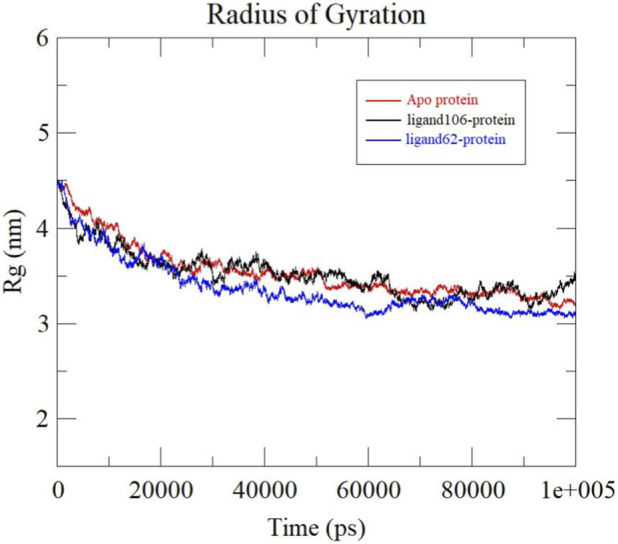
Rg plots obtained for Apo protein (red), ActA-Xambioona complex (black) and ActA-Licoisoflavone B complex (blue).

Rg is an indication of the structural compactness of the protein. By calculating the atom distribution around the mass center, the compactness of the system is evaluated. Rg value also determines if the complex unfolds or folds steadily over the course of the simulation (Sharma et al., 2020; Thirumal Kumar et al., 2017; Apicella et al., 2017). The Rg plots demonstrating the compactness of Apo-protein, ActA-Xambioona complex and ActA-Licoisoflavone B complex are depicted in Figure 12. Apo protein showcased an average Rg value of 3.33 ± 0.08 nm. ActA-ligand106 and ActA-ligand62 displayed Rg values of 3.36 ± 0.08 nm and 3.22 ± 0.06 nm, respectively. Systems with lower Rg values are considered to be more compact than those with higher Rg. In this study, highest Rg value was recorded for the ActA-ligand106 complex followed by the apo protein. The ActA-Licoisoflavone B complex produced the lowest Rg value suggesting that this complex is more compact in comparison to the other systems under study. The ActA-Xambioona complex is the least compact of the three systems.

#### 3.5.4 Hydrogen bond analysis

**FIGURE 13:**
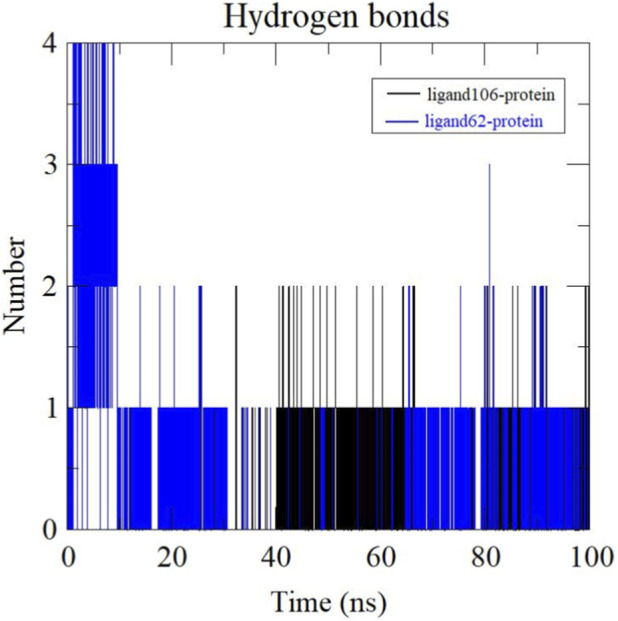
The number of hydrogen bonds formed between the ActA-Xambioona complex (black) and the ActA-Licoisoflavone B complex (Blue).

One of the key factors in determining the specificity of ligand binding is hydrogen bonding. Other than playing a vital role in protein-ligand interactions, it can also affect the affinity, adsorption and metabolism of the drug (Patil et al., 2010). The number of hydrogen bonds formed during each 100 ns trajectory were evaluated for both ActA-Xambioona and ActA-Licoisoflavone B complexes (Figure 13). A maximum of 2 hydrogen bonds were observed for the ActA-ligand106 complex by the end of 100 ns although no hydrogen bonds appeared until 15 ns. In the case of ActA-ligand62 complex, 4 hydrogen bonds were formed initially but towards the end of simulation, only 1 hydrogen bond was retained. The increase in the number of hydrogen bonds at the start of simulation can be explained by the initial structural relaxation and conformational change, which enables the ligand to scan a larger interaction landscape in the binding pocket. The number of hydrogen bonds reached a point of stability at one active interaction as the simulation continued. This decrease implies that not all hydrogen bonds formed in the first place will be stable under dynamic conditions, some hydrogen bonds were short-lived and disappeared during equilibration.

### 3.6 Estimation of binding free energy

**FIGURE 14:**
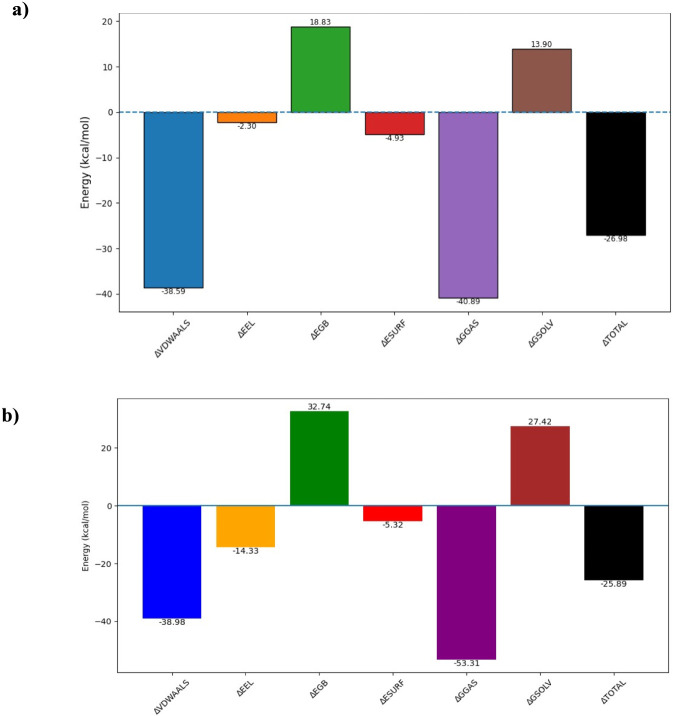
Energy contributions and overall binding free energies of **(a)** ActA-ligand62 and **(b)** ActA-ligand106 complexes.

MM-PBSA was performed for the 100 ns MD simulation in order to determine the binding free energies of ActA-ligand106 and ActA-ligand62 complexes. The components of energy that were computed were the van der Waals molecular mechanics energy (ΔE_VDWAAL_), electrostatic molecular mechanics energy (ΔE_EEL_), polar contribution to the solvation energy (ΔE_GB_), solvent accessible surface area (ΔE_SURF_), total gas phase molecular mechanics energy (ΔG_GAS_) and total solvation energy (ΔG_SOLV_). The determined ΔG_bind_ indicated that the ActA-ligand62 complex had a more favorable binding free energy value of −26.98 kcal/mol than ActA-ligand106 complex (−25.89 kcal/mol). Hence, the ActA-Licoisoflavone B complex was more stable and had energetically favorable interactions based on the generated energy data. Figures 14a,b illustrates the individual energy contributions and total binding free energies of ActA-ligand62 and ActA-ligand106 complexes.

## 4 Discussion

The increasing burden of antimicrobial resistance in *Listeria monocytogenes* has seriously compromised the effectiveness of traditional antibiotics, especially those that act on crucial pathways of bacterial survival. The selective pressure exerted by antimicrobial agents has led to the emergence of resistant strains rapidly through mechanisms like biofilm formation, horizontal gene transfer and stress adaptations. As a result, there appears to be a growing interest in alternative treatment methods that lower pathogenicity without affecting the host microbiota. One such approach is focusing the virulence proteins of this disease-causing bacteria that play various key functions in the survival and pathogenicity of *Listeria* potentially paving the way to an effective treatment strategy compared to the conventional therapeutics (Rasko and Sperandio, 2010). By intervening the pathogenicity of *Listeria* instead of viability, aiming ActA offers a novel strategy that could be unique from standard antibiotic therapy. The ability of ActA to initiate actin polymerization thereby promoting bacterial movement and spread of infection throughout the host cell, allows the pathogen to avoid exposure to antibiotics and immune defenses. By hindering this transmission mechanism through ActA inhibition, the pathogen can effectively be confined to the host cells alone wherein the immune clearance is boosted. When compared to traditional antibiotic treatment that are directed at key growth pathways, this strategy could lower the probability of resistance emergence as this method does not involve significant antimicrobial pressure.

In this study, Liquorice-derived compounds were explored as potential inhibitors of an essential virulence protein ActA of the pathogen, through an *in silico* strategy. Since no experimentally determined structure was available for ActA protein, two modeling servers AlphaFold Colab and RoseTTAFold were used to increase the reliability of the virulence protein structure. In order to achieve structural reliability, extensive validation was conducted to understand the stereochemical quality, non-bonded atomic interactions and the overall structural plausibility of the obtained protein models. These validation steps guaranteed that the protein models displayed characteristics that aligned with the experimentally resolved structures and provided a structural foundation to further *in silico* approaches similar to previously published reports (Chao et al., 2024). Subsequent virtual screening of the Liquorice phytocompounds against the 2 predicted ActA models revealed AlphaFold Colab model to have better binding affinity profiles than RoseTTAFold model. The AlphaFold model was favored for subsequent interaction analysis since this model offered a framework for ligand binding that was more chemically and structurally consistent. Xambioona (−10.4 kcal/mol) and Licoisoflavone B (−8.7 kcal/mol) were the topmost compounds discovered to have the highest binding affinity to the virulence protein. Virtual screening is a rationale and efficient method to identify bioactive natural compounds with favorable interaction profiles. Previous literature has reported a wide variety of antimicrobial activities of plant derived phytocompounds, with certain classes having the ability to modulate virulence factors involved in quorum sensing, biofilms, motility or toxin expression (Silva et al., 2016). Both the compounds, Xambioona and Licoisoflavone B formed stabilizing hydrophobic interactions with the ActA protein contributing to the overall stability of the respective protein-ligand complexes. In addition, Licoisoflavone B formed hydrogen bonds with the virulence protein that possibly contributed to the overall pose stability and specificity. All the aforementioned interactions indicate that the compounds could possibly disrupt the functional area associated with the virulence protein activity. Beyond virtual screening, this work also utilized molecular dynamics (MD) simulations to study the dynamic behavior of the protein-ligand complexes under similar physiological conditions making it possible to analyze the structural stability and the persistence of interactions over a timeframe of 100 ns. The ActA-Licoisoflavone B complex exhibited reduced deviations and a more stable interaction profile than the ActA-Xambioona complex during the 100 ns time period despite Xambioona’s superior binding affinity with the protein. MD simulations include conformational flexibility and solvent effects, which makes the interactions of the ligands and the proteins more realistic. The higher stability of ActA-Licoisoflavone B complex demonstrates that dynamic interaction persistence and not merely initial binding affinity is a significant factor in determining complex stability. These computational findings resonate with other reports in which *in silico* screening and simulation of phytochemicals against virulence targets generated high-priority leads to further research. For example, in a previous study, 20 anti-biofilm compounds were screened and subjected to MD simulations as a primary approach to discover natural compounds with a potential inhibitory activity against *Pseudomonas aeruginosa* (Behera et al., 2023). Thus, based on the results of this *in silico* work, Licoisoflavone B was determined to be the most effective phytocompound that could be targeted as a potential antibacterial agent against ActA protein. However, experimental validations are required to confirm the computational results discussed in this research.

Overall, this study highlights the significance of integrating natural product research with computational biology in the quest for novel therapeutic approaches. The identification of Licoisoflavone B offers a foundation for forthcoming biochemical assessments and various other experimental initiatives aiming the development of anti-virulence treatment regimens that could contribute to sustained antimicrobial resistance control.

## 5 Conclusion

The best compounds identified from this work will be the subject of future investigations that will focus on the *in vivo/in vitro* validations.

## Data Availability

The original contributions presented in the study are included in the article/Supplementary Material, further inquiries can be directed to the corresponding author.
