# Supplementary material for: In silico identification of phytocompounds derived from Glycyrrhiza glabra as potential inhibitors of actin assembly-inducing protein in Listeria monocytogenes: a virtual screening and molecular dynamics study
Source: Front Bioinform. 2026 Apr 28;6:1822250. doi: 10.3389/fbinf.2026.1822250 (PMC13161068; doi:10.3389/fbinf.2026.1822250)
Supplement: Supplementary file 1 [file Table1.docx]

**S1. VIRTUAL SCREENING ANALYSIS OF LICORICE PHYTOCOMPOUNDS AGAINST ACTA PROTEIN (AlphaFold Colab model)**

| **SL.NO.** | **LIGANDS** | **BINDING ENERGY** |
| --- | --- | --- |
| 1. | [Genistein](https://cb.imsc.res.in/imppat/phytochemical-detailedpage/IMPHY004643) | -7.0 |
| 2. | [Galangin](https://cb.imsc.res.in/imppat/phytochemical-detailedpage/IMPHY005434) | -7.8 |
| 3. | [Hydroxywighteone](https://cb.imsc.res.in/imppat/phytochemical-detailedpage/IMPHY005925) | -7.5 |
| 4. | [Pinocembrin](https://cb.imsc.res.in/imppat/phytochemical-detailedpage/IMPHY007196) | -7.3 |
| 5. | [Naringetol](https://cb.imsc.res.in/imppat/phytochemical-detailedpage/IMPHY010550)(Naringenin) | -7.0 |
| 6. | [Glabranin](https://cb.imsc.res.in/imppat/phytochemical-detailedpage/IMPHY010555) | -7.2 |
| 7. | [Methoxsalen](https://cb.imsc.res.in/imppat/phytochemical-detailedpage/IMPHY003037) | -6.1 |
| 8. | [Kaempferol](https://cb.imsc.res.in/imppat/phytochemical-detailedpage/IMPHY004388) | -7.1 |
| 9. | [Quercetin](https://cb.imsc.res.in/imppat/phytochemical-detailedpage/IMPHY004619) | -7.0 |
| 10. | [Bergapten](https://cb.imsc.res.in/imppat/phytochemical-detailedpage/IMPHY005428) | -6.4 |
| 11. | [Isovitexin](https://cb.imsc.res.in/imppat/phytochemical-detailedpage/IMPHY008689) | -7.6 |
| 12. | [Lutein](https://cb.imsc.res.in/imppat/phytochemical-detailedpage/IMPHY011620) | -7.3 |
| 13. | [beta-Carotene](https://cb.imsc.res.in/imppat/phytochemical-detailedpage/IMPHY011707) | -7.1 |
| 14. | [Linalool](https://cb.imsc.res.in/imppat/phytochemical-detailedpage/IMPHY012058) | -5.0 |
| 15. | [Isoquercitrin](https://cb.imsc.res.in/imppat/phytochemical-detailedpage/IMPHY012721) | -7.3 |
| 16. | [beta-Sitosterol](https://cb.imsc.res.in/imppat/phytochemical-detailedpage/IMPHY014836) | -7.0 |
| 17. | [Dehydroepiandrosterone](https://cb.imsc.res.in/imppat/phytochemical-detailedpage/IMPHY016827) | -6.9 |
| 18. | [Pyrazine, 1,4-dioxide](https://cb.imsc.res.in/imppat/phytochemical-detailedpage/IMPHY016835) | -3.7 |
| 19. | [4-Aminopyridine](https://cb.imsc.res.in/imppat/phytochemical-detailedpage/IMPHY017165) | -3.6 |
| 20. | [4-(2-Aminopropyl)phenol](https://cb.imsc.res.in/imppat/phytochemical-detailedpage/IMPHY017284) [hydroxyamphetamine] | -5.1 |
| 21. | [Licoflavone B](https://cb.imsc.res.in/imppat/phytochemical-detailedpage/IMPHY000013) | -7.2 |
| 22. | Myristic acid | -4.8 |
| 23. | [Glabroisoflavanone A](https://cb.imsc.res.in/imppat/phytochemical-detailedpage/IMPHY000151) | -7.6 |
| 24. | Paeonol | -6.8 |
| 25. | [3-Hydroxyglabrol](https://cb.imsc.res.in/imppat/phytochemical-detailedpage/IMPHY000375) | -6.4 |
| 26. | [7-Methoxycoumarin](https://cb.imsc.res.in/imppat/phytochemical-detailedpage/IMPHY000688) | -5.4 |
| 27. | [Glabrene](https://cb.imsc.res.in/imppat/phytochemical-detailedpage/IMPHY000852) | -7.9 |
| 28. | [Glycyrin](https://cb.imsc.res.in/imppat/phytochemical-detailedpage/IMPHY000864) | -7.7 |
| 29. | [Shinpterocarpin](https://cb.imsc.res.in/imppat/phytochemical-detailedpage/IMPHY001239) | -7.3 |
| 30. | [Carvacrol](https://cb.imsc.res.in/imppat/phytochemical-detailedpage/IMPHY001246) | -6.0 |
| 31. | [Hispaglabridin B](https://cb.imsc.res.in/imppat/phytochemical-detailedpage/IMPHY001500) | -7.0 |
| 32. | [Isoglabrolide](https://cb.imsc.res.in/imppat/phytochemical-detailedpage/IMPHY001586) | -7.1 |
| 33. | [Glabroisoflavanone B](https://cb.imsc.res.in/imppat/phytochemical-detailedpage/IMPHY001793) | -7.3 |
| 34. | [Glabrocoumarin](https://cb.imsc.res.in/imppat/phytochemical-detailedpage/IMPHY001798) | -8.1 |
| 35. | [Liqcoumarin](https://cb.imsc.res.in/imppat/phytochemical-detailedpage/IMPHY001806) | -6.0 |
| 36. | Glabrol | -7.6 |
| 37. | [Liquiritigenin](https://cb.imsc.res.in/imppat/phytochemical-detailedpage/IMPHY001869) | -7.2 |
| 38. | [4-Methylcoumarin](https://cb.imsc.res.in/imppat/phytochemical-detailedpage/IMPHY001923) | -6.4 |
| 39. | [Glychionide A](https://cb.imsc.res.in/imppat/phytochemical-detailedpage/IMPHY001944) | -6.8 |
| 40. | [2,3,5,6-Tetramethylpyrazine](https://cb.imsc.res.in/imppat/phytochemical-detailedpage/IMPHY002803) | -4.5 |
| 41. | [Piperitenone](https://cb.imsc.res.in/imppat/phytochemical-detailedpage/IMPHY003296) | -5.8 |
| 42. | [Eugenol](https://cb.imsc.res.in/imppat/phytochemical-detailedpage/IMPHY003536) | -5.6 |
| 43. | [D-Glucuronic Acid](https://cb.imsc.res.in/imppat/phytochemical-detailedpage/IMPHY004235) | -4.6 |
| 44. | [Pratol](https://cb.imsc.res.in/imppat/phytochemical-detailedpage/IMPHY004344) | -7.2 |
| 45. | [Kumatakenin](https://cb.imsc.res.in/imppat/phytochemical-detailedpage/IMPHY004360) (Jaranol) | -6.9 |
| 46. | [Licoagrone](https://cb.imsc.res.in/imppat/phytochemical-detailedpage/IMPHY004362) | -7.0 |
| 47. | [Licoricone](https://cb.imsc.res.in/imppat/phytochemical-detailedpage/IMPHY004365) | -7.2 |
| 48. | [Safrole](https://cb.imsc.res.in/imppat/phytochemical-detailedpage/IMPHY004549) | -5.0 |
| 49. | [Texasin](https://cb.imsc.res.in/imppat/phytochemical-detailedpage/IMPHY004586) | -6.5 |
| 50. | [Licoisoflavone A](https://cb.imsc.res.in/imppat/phytochemical-detailedpage/IMPHY004595) | -7.6 |
| 51. | [Prunetin](https://cb.imsc.res.in/imppat/phytochemical-detailedpage/IMPHY004601) | -6.8 |
| 52. | [Apigenin](https://cb.imsc.res.in/imppat/phytochemical-detailedpage/IMPHY004661) | -6.9 |
| 53. | Glyzarin | -7.6 |
| 54. | [Liquiritin](https://cb.imsc.res.in/imppat/phytochemical-detailedpage/IMPHY004759) | -8.5 |
| 55. | [Hispaglabridin A](https://cb.imsc.res.in/imppat/phytochemical-detailedpage/IMPHY004868) | -7.1 |
| 56. | [Glabrone](https://cb.imsc.res.in/imppat/phytochemical-detailedpage/IMPHY004974) | -7.7 |
| 57. | [Glycyrrhisoflavone](https://cb.imsc.res.in/imppat/phytochemical-detailedpage/IMPHY004988) | -6.9 |
| 58. | Glyzaglabrin | -7.4 |
| 59. | Umbelliferone | -6.0 |
| 60. | [Neoisoliquiritin](https://cb.imsc.res.in/imppat/phytochemical-detailedpage/IMPHY005669) | -7.3 |
| 61. | [Isoangustone A](https://cb.imsc.res.in/imppat/phytochemical-detailedpage/IMPHY005762) | -6.8 |
| 62. | [Licoisoflavone B](https://cb.imsc.res.in/imppat/phytochemical-detailedpage/IMPHY005876) | -8.7 |
| 63. | [Licoflavonol](https://cb.imsc.res.in/imppat/phytochemical-detailedpage/IMPHY005893) | -7.8 |
| 64. | [Glabrolide](https://cb.imsc.res.in/imppat/phytochemical-detailedpage/IMPHY006740) | -7.7 |
| 65. | [Estragole](https://cb.imsc.res.in/imppat/phytochemical-detailedpage/IMPHY006944) | -5.1 |
| 66. | [Eicosane](https://cb.imsc.res.in/imppat/phytochemical-detailedpage/IMPHY006951) | -5.1 |
| 67. | [Licoriphenone](https://cb.imsc.res.in/imppat/phytochemical-detailedpage/IMPHY007958) | -7.3 |
| 68. | [Licoagrodin](https://cb.imsc.res.in/imppat/phytochemical-detailedpage/IMPHY008216) | -7.3 |
| 69. | [Formononetin](https://cb.imsc.res.in/imppat/phytochemical-detailedpage/IMPHY009035) | -6.7 |
| 70. | [Shinflavanone](https://cb.imsc.res.in/imppat/phytochemical-detailedpage/IMPHY009114) | -6.9 |
| 71. | [Glabridin](https://cb.imsc.res.in/imppat/phytochemical-detailedpage/IMPHY009376) | -8.3 |
| 72. | [4-Carvomenthenol](https://cb.imsc.res.in/imppat/phytochemical-detailedpage/IMPHY011396) | -5.6 |
| 73. | [Anethole](https://cb.imsc.res.in/imppat/phytochemical-detailedpage/IMPHY011763) | -5.3 |
| 74. | [Pulegone](https://cb.imsc.res.in/imppat/phytochemical-detailedpage/IMPHY011884) | -5.7 |
| 75. | [alpha-Terpineol](https://cb.imsc.res.in/imppat/phytochemical-detailedpage/IMPHY012160) | -6.0 |
| 76. | [Ambrettolide](https://cb.imsc.res.in/imppat/phytochemical-detailedpage/IMPHY012826) | -6.1 |
| 77. | [Licuroside](https://cb.imsc.res.in/imppat/phytochemical-detailedpage/IMPHY012954) | -7.7 |
| 78. | [Kanzonol R](https://cb.imsc.res.in/imppat/phytochemical-detailedpage/IMPHY013718) | -7.8 |
| 79. | [Stigmasterol](https://cb.imsc.res.in/imppat/phytochemical-detailedpage/IMPHY014842) | -6.5 |
| 80. | [Enoxolone](https://cb.imsc.res.in/imppat/phytochemical-detailedpage/IMPHY014926) | -6.4 |
| 81. | Glyasperin D | -6.8 |
| 82. | 1-Methoxyphaseollidin | -8.2 |
| 83. | Licoricidin | -7.3 |
| 84. | [Gancaonin R](https://cb.imsc.res.in/imppat/phytochemical-detailedpage/IMPHY000791) | -7.7 |
| 85. | [8-Prenylnaringenin](https://cb.imsc.res.in/imppat/phytochemical-detailedpage/IMPHY000819) | -7.7 |
| 86. | Glyinflanin B | -7.3 |
| 87. | [Licoagrodione](https://cb.imsc.res.in/imppat/phytochemical-detailedpage/IMPHY001244) | -7.6 |
| 88. | Medicarpin | -7.2 |
| 89. | Apiin | -6.3 |
| 90. | Echinatin | -6.7 |
| 91. | [Lavandulol](https://cb.imsc.res.in/imppat/phytochemical-detailedpage/IMPHY006428) | -5.2 |
| 92. | [Magnolol](https://cb.imsc.res.in/imppat/phytochemical-detailedpage/IMPHY006690) | -6.5 |
| 93. | [Ononin](https://cb.imsc.res.in/imppat/phytochemical-detailedpage/IMPHY006894) | -7.5 |
| 94. | [Maltol](https://cb.imsc.res.in/imppat/phytochemical-detailedpage/IMPHY007058) | -4.4 |
| 95. | [Hemileiocarpin](https://cb.imsc.res.in/imppat/phytochemical-detailedpage/IMPHY008064) | -7.2 |
| 96. | [Glyyunnansapogenin B](https://cb.imsc.res.in/imppat/phytochemical-detailedpage/IMPHY009680) | -6.5 |
| 97. | [gamma-Caprolactone](https://cb.imsc.res.in/imppat/phytochemical-detailedpage/IMPHY009824) | -4.3 |
| 98. | [Guaiacol](https://cb.imsc.res.in/imppat/phytochemical-detailedpage/IMPHY011409) | -4.6 |
| 99. | [Thujone](https://cb.imsc.res.in/imppat/phytochemical-detailedpage/IMPHY011901) | -5.5 |
| 100. | [Anabasine](https://cb.imsc.res.in/imppat/phytochemical-detailedpage/IMPHY012035) | -5.5 |
| 101. | [beta-Amyrin](https://cb.imsc.res.in/imppat/phytochemical-detailedpage/IMPHY012223) | -7.3 |
| 102. | [Fenchone](https://cb.imsc.res.in/imppat/phytochemical-detailedpage/IMPHY013836) | -5.6 |
| 103. | [Astragalin](https://cb.imsc.res.in/imppat/phytochemical-detailedpage/IMPHY014824) | -6.1 |
| 104. | [Licoagrocarpin](https://cb.imsc.res.in/imppat/phytochemical-detailedpage/IMPHY015792) | -7.1 |
| 105. | [Isomucronulatol](https://cb.imsc.res.in/imppat/phytochemical-detailedpage/IMPHY015849) | -6.7 |
| 106. | [Xambioona](https://cb.imsc.res.in/imppat/phytochemical-detailedpage/IMPHY015988) | -10.4 |

**S2. VIRTUAL SCREENING ANALYSIS OF LICORICE PHYTOCOMPOUNDS AGAINST ACTA PROTEIN (RoseTTAFold model)**

| **SL.NO.** | **LIGANDS** | **BINDING ENERGY** |
| --- | --- | --- |
| 1. | [Genistein](https://cb.imsc.res.in/imppat/phytochemical-detailedpage/IMPHY004643) | -5.4 |
| 2. | [Galangin](https://cb.imsc.res.in/imppat/phytochemical-detailedpage/IMPHY005434) | -6.4 |
| 3. | [Hydroxywighteone](https://cb.imsc.res.in/imppat/phytochemical-detailedpage/IMPHY005925) | -5.2 |
| 4. | [Pinocembrin](https://cb.imsc.res.in/imppat/phytochemical-detailedpage/IMPHY007196) | -4.9 |
| 5. | [Naringetol](https://cb.imsc.res.in/imppat/phytochemical-detailedpage/IMPHY010550)(Naringenin) | -6.1 |
| 6. | [Glabranin](https://cb.imsc.res.in/imppat/phytochemical-detailedpage/IMPHY010555) | -4.8 |
| 7. | [Methoxsalen](https://cb.imsc.res.in/imppat/phytochemical-detailedpage/IMPHY003037) | -5.1 |
| 8. | [Kaempferol](https://cb.imsc.res.in/imppat/phytochemical-detailedpage/IMPHY004388) | -5.1 |
| 9. | [Quercetin](https://cb.imsc.res.in/imppat/phytochemical-detailedpage/IMPHY004619) | -5.4 |
| 10. | [Bergapten](https://cb.imsc.res.in/imppat/phytochemical-detailedpage/IMPHY005428) | -5.2 |
| 11. | [Isovitexin](https://cb.imsc.res.in/imppat/phytochemical-detailedpage/IMPHY008689) | -4.9 |
| 12. | [Lutein](https://cb.imsc.res.in/imppat/phytochemical-detailedpage/IMPHY011620) | -5.3 |
| 13. | [beta-Carotene](https://cb.imsc.res.in/imppat/phytochemical-detailedpage/IMPHY011707) | -5.4 |
| 14. | [Linalool](https://cb.imsc.res.in/imppat/phytochemical-detailedpage/IMPHY012058) | -3.5 |
| 15. | [Isoquercitrin](https://cb.imsc.res.in/imppat/phytochemical-detailedpage/IMPHY012721) | -5.6 |
| 16. | [beta-Sitosterol](https://cb.imsc.res.in/imppat/phytochemical-detailedpage/IMPHY014836) | -5.1 |
| 17. | [Dehydroepiandrosterone](https://cb.imsc.res.in/imppat/phytochemical-detailedpage/IMPHY016827) | -4.1 |
| 18. | [Pyrazine, 1,4-dioxide](https://cb.imsc.res.in/imppat/phytochemical-detailedpage/IMPHY016835) | -3.3 |
| 19. | [4-Aminopyridine](https://cb.imsc.res.in/imppat/phytochemical-detailedpage/IMPHY017165) | -3.3 |
| 20. | [4-(2-Aminopropyl)phenol](https://cb.imsc.res.in/imppat/phytochemical-detailedpage/IMPHY017284) [hydroxyamphetamine] | -4.5 |
| 21. | [Licoflavone B](https://cb.imsc.res.in/imppat/phytochemical-detailedpage/IMPHY000013) | -5.5 |
| 22. | Myristic acid | -3.2 |
| 23. | [Glabroisoflavanone A](https://cb.imsc.res.in/imppat/phytochemical-detailedpage/IMPHY000151) | -4.0 |
| 24. | Paeonol | -5.3 |
| 25. | [3-Hydroxyglabrol](https://cb.imsc.res.in/imppat/phytochemical-detailedpage/IMPHY000375) | -5.2 |
| 26. | [7-Methoxycoumarin](https://cb.imsc.res.in/imppat/phytochemical-detailedpage/IMPHY000688) | -5.1 |
| 27. | [Glabrene](https://cb.imsc.res.in/imppat/phytochemical-detailedpage/IMPHY000852) | -6.6 |
| 28. | [Glycyrin](https://cb.imsc.res.in/imppat/phytochemical-detailedpage/IMPHY000864) | -5.7 |
| 29. | [Shinpterocarpin](https://cb.imsc.res.in/imppat/phytochemical-detailedpage/IMPHY001239) | -5.4 |
| 30. | [Carvacrol](https://cb.imsc.res.in/imppat/phytochemical-detailedpage/IMPHY001246) | -4.8 |
| 31. | [Hispaglabridin B](https://cb.imsc.res.in/imppat/phytochemical-detailedpage/IMPHY001500) | -5.0 |
| 32. | [Isoglabrolide](https://cb.imsc.res.in/imppat/phytochemical-detailedpage/IMPHY001586) | -5.2 |
| 33. | [Glabroisoflavanone B](https://cb.imsc.res.in/imppat/phytochemical-detailedpage/IMPHY001793) | -5.7 |
| 34. | [Glabrocoumarin](https://cb.imsc.res.in/imppat/phytochemical-detailedpage/IMPHY001798) | -6.5 |
| 35. | [Liqcoumarin](https://cb.imsc.res.in/imppat/phytochemical-detailedpage/IMPHY001806) | -5.0 |
| 36. | Glabrol | -5.0 |
| 37. | [Liquiritigenin](https://cb.imsc.res.in/imppat/phytochemical-detailedpage/IMPHY001869) | -5.7 |
| 38. | [4-Methylcoumarin](https://cb.imsc.res.in/imppat/phytochemical-detailedpage/IMPHY001923) | -5.1 |
| 39. | [Glychionide A](https://cb.imsc.res.in/imppat/phytochemical-detailedpage/IMPHY001944) | -5.2 |
| 40. | [2,3,5,6-Tetramethylpyrazine](https://cb.imsc.res.in/imppat/phytochemical-detailedpage/IMPHY002803) | -3.7 |
| 41. | [Piperitenone](https://cb.imsc.res.in/imppat/phytochemical-detailedpage/IMPHY003296) | -4.9 |
| 42. | [Eugenol](https://cb.imsc.res.in/imppat/phytochemical-detailedpage/IMPHY003536) | -4.2 |
| 43. | [D-Glucuronic Acid](https://cb.imsc.res.in/imppat/phytochemical-detailedpage/IMPHY004235) | -4.4 |
| 44. | [Pratol](https://cb.imsc.res.in/imppat/phytochemical-detailedpage/IMPHY004344) | -5.3 |
| 45. | [Kumatakenin](https://cb.imsc.res.in/imppat/phytochemical-detailedpage/IMPHY004360) (Jaranol) | -5.4 |
| 46. | [Licoagrone](https://cb.imsc.res.in/imppat/phytochemical-detailedpage/IMPHY004362) | -5.0 |
| 47. | [Licoricone](https://cb.imsc.res.in/imppat/phytochemical-detailedpage/IMPHY004365) | -5.5 |
| 48. | [Safrole](https://cb.imsc.res.in/imppat/phytochemical-detailedpage/IMPHY004549) | -4.5 |
| 49. | [Texasin](https://cb.imsc.res.in/imppat/phytochemical-detailedpage/IMPHY004586) | -5.4 |
| 50. | [Licoisoflavone A](https://cb.imsc.res.in/imppat/phytochemical-detailedpage/IMPHY004595) | -5.0 |
| 51. | [Prunetin](https://cb.imsc.res.in/imppat/phytochemical-detailedpage/IMPHY004601) | -5.7 |
| 52. | [Apigenin](https://cb.imsc.res.in/imppat/phytochemical-detailedpage/IMPHY004661) | -4.8 |
| 53. | Glyzarin | -5.5 |
| 54. | [Liquiritin](https://cb.imsc.res.in/imppat/phytochemical-detailedpage/IMPHY004759) | -6.2 |
| 55. | [Hispaglabridin A](https://cb.imsc.res.in/imppat/phytochemical-detailedpage/IMPHY004868) | -5.1 |
| 56. | [Glabrone](https://cb.imsc.res.in/imppat/phytochemical-detailedpage/IMPHY004974) | -5.4 |
| 57. | [Glycyrrhisoflavone](https://cb.imsc.res.in/imppat/phytochemical-detailedpage/IMPHY004988) | -5.0 |
| 58. | Glyzaglabrin | -5.7 |
| 59. | Umbelliferone | -4.6 |
| 60. | [Neoisoliquiritin](https://cb.imsc.res.in/imppat/phytochemical-detailedpage/IMPHY005669) | -4.9 |
| 61. | [Isoangustone A](https://cb.imsc.res.in/imppat/phytochemical-detailedpage/IMPHY005762) | -5.1 |
| 62. | [Licoisoflavone B](https://cb.imsc.res.in/imppat/phytochemical-detailedpage/IMPHY005876) | -6.7 |
| 63. | [Licoflavonol](https://cb.imsc.res.in/imppat/phytochemical-detailedpage/IMPHY005893) | -6.4 |
| 64. | [Glabrolide](https://cb.imsc.res.in/imppat/phytochemical-detailedpage/IMPHY006740) | -4.8 |
| 65. | [Estragole](https://cb.imsc.res.in/imppat/phytochemical-detailedpage/IMPHY006944) | -4.2 |
| 66. | [Eicosane](https://cb.imsc.res.in/imppat/phytochemical-detailedpage/IMPHY006951) | -3.0 |
| 67. | [Licoriphenone](https://cb.imsc.res.in/imppat/phytochemical-detailedpage/IMPHY007958) | -5.6 |
| 68. | [Licoagrodin](https://cb.imsc.res.in/imppat/phytochemical-detailedpage/IMPHY008216) | -5.7 |
| 69. | [Formononetin](https://cb.imsc.res.in/imppat/phytochemical-detailedpage/IMPHY009035) | -5.0 |
| 70. | [Shinflavanone](https://cb.imsc.res.in/imppat/phytochemical-detailedpage/IMPHY009114) | -5.3 |
| 71. | [Glabridin](https://cb.imsc.res.in/imppat/phytochemical-detailedpage/IMPHY009376) | -7.0 |
| 72. | [4-Carvomenthenol](https://cb.imsc.res.in/imppat/phytochemical-detailedpage/IMPHY011396) | -4.8 |
| 73. | [Anethole](https://cb.imsc.res.in/imppat/phytochemical-detailedpage/IMPHY011763) | -4.5 |
| 74. | [Pulegone](https://cb.imsc.res.in/imppat/phytochemical-detailedpage/IMPHY011884) | -4.5 |
| 75. | [alpha-Terpineol](https://cb.imsc.res.in/imppat/phytochemical-detailedpage/IMPHY012160) | -4.8 |
| 76. | [Ambrettolide](https://cb.imsc.res.in/imppat/phytochemical-detailedpage/IMPHY012826) | -5.0 |
| 77. | [Licuroside](https://cb.imsc.res.in/imppat/phytochemical-detailedpage/IMPHY012954) | -5.7 |
| 78. | [Kanzonol R](https://cb.imsc.res.in/imppat/phytochemical-detailedpage/IMPHY013718) | -6.4 |
| 79. | [Stigmasterol](https://cb.imsc.res.in/imppat/phytochemical-detailedpage/IMPHY014842) | -5.2 |
| 80. | [Enoxolone](https://cb.imsc.res.in/imppat/phytochemical-detailedpage/IMPHY014926) | -5.7 |
| 81. | Glyasperin D | -5.2 |
| 82. | 1-Methoxyphaseollidin | -5.8 |
| 83. | Licoricidin | -5.4 |
| 84. | [Gancaonin R](https://cb.imsc.res.in/imppat/phytochemical-detailedpage/IMPHY000791) | -5.1 |
| 85. | [8-Prenylnaringenin](https://cb.imsc.res.in/imppat/phytochemical-detailedpage/IMPHY000819) | -4.9 |
| 86. | Glyinflanin B | -4.8 |
| 87. | [Licoagrodione](https://cb.imsc.res.in/imppat/phytochemical-detailedpage/IMPHY001244) | -4.8 |
| 88. | Medicarpin | -5.1 |
| 89. | Apiin | -5.7 |
| 90. | Echinatin | -5.6 |
| 91. | [Lavandulol](https://cb.imsc.res.in/imppat/phytochemical-detailedpage/IMPHY006428) | -4.1 |
| 92. | [Magnolol](https://cb.imsc.res.in/imppat/phytochemical-detailedpage/IMPHY006690) | -5.2 |
| 93. | [Ononin](https://cb.imsc.res.in/imppat/phytochemical-detailedpage/IMPHY006894) | -5.0 |
| 94. | [Maltol](https://cb.imsc.res.in/imppat/phytochemical-detailedpage/IMPHY007058) | -4.0 |
| 95. | [Hemileiocarpin](https://cb.imsc.res.in/imppat/phytochemical-detailedpage/IMPHY008064) | -4.9 |
| 96. | [Glyyunnansapogenin B](https://cb.imsc.res.in/imppat/phytochemical-detailedpage/IMPHY009680) | -5.6 |
| 97. | [gamma-Caprolactone](https://cb.imsc.res.in/imppat/phytochemical-detailedpage/IMPHY009824) | -3.6 |
| 98. | [Guaiacol](https://cb.imsc.res.in/imppat/phytochemical-detailedpage/IMPHY011409) | -4.1 |
| 99. | [Thujone](https://cb.imsc.res.in/imppat/phytochemical-detailedpage/IMPHY011901) | -4.1 |
| 100. | [Anabasine](https://cb.imsc.res.in/imppat/phytochemical-detailedpage/IMPHY012035) | -4.5 |
| 101. | [beta-Amyrin](https://cb.imsc.res.in/imppat/phytochemical-detailedpage/IMPHY012223) | -5.1 |
| 102. | [Fenchone](https://cb.imsc.res.in/imppat/phytochemical-detailedpage/IMPHY013836) | -4.4 |
| 103. | [Astragalin](https://cb.imsc.res.in/imppat/phytochemical-detailedpage/IMPHY014824) | -5.3 |
| 104. | [Licoagrocarpin](https://cb.imsc.res.in/imppat/phytochemical-detailedpage/IMPHY015792) | -5.4 |
| 105. | [Isomucronulatol](https://cb.imsc.res.in/imppat/phytochemical-detailedpage/IMPHY015849) | -5.6 |
| 106. | [Xambioona](https://cb.imsc.res.in/imppat/phytochemical-detailedpage/IMPHY015988) | -8.0 |
